# Assessing the impacts of total liquid ventilation on left ventricular diastolic function in a model of neonatal respiratory distress syndrome

**DOI:** 10.1371/journal.pone.0191885

**Published:** 2018-01-29

**Authors:** Michaël Sage, Mathieu Nadeau, Claudia Forand-Choinière, Julien Mousseau, Jonathan Vandamme, Claire Berger, Jean-Sébastien Tremblay-Roy, Renaud Tissier, Philippe Micheau, Étienne Fortin-Pellerin

**Affiliations:** 1 Department of Pediatrics and Department of Pharmacology and Physiology, Université de Sherbrooke, Sherbrooke, Québec, Canada; 2 Department of Mechanical Engineering, Université de Sherbrooke, Sherbrooke, Québec, Canada; 3 Department of Medicine, Université de Poitiers, Poitiers, France; 4 Inserm, Unité 955, Equipe 03, École Nationale Vétérinaire d’Alfort, Université Paris-Est Créteil, Paris, France; Scuola Superiore Sant'Anna, ITALY

## Abstract

**Background:**

Filling the lung with dense liquid perfluorocarbons during total liquid ventilation (TLV) might compress the myocardium, a plausible explanation for the instability occasionally reported with this technique. Our objective is to assess the impacts of TLV on the cardiovascular system, particularly left ventricular diastolic function, in an ovine model of neonatal respiratory distress syndrome.

**Method:**

Eight newborns lambs, 3.0 ± 0.4 days (3.2 ± 0.3kg) were used in this crossover experimental study. Animals were intubated, anesthetized and paralyzed. Catheters were inserted in the femoral and pulmonary arteries. A high-fidelity pressure catheter was inserted into the left ventricle. Surfactant deficiency was induced by repeated lung lavages with normal saline. TLV was then conducted for 2 hours using a liquid ventilator prototype. Thoracic echocardiography and cardiac output assessment by thermodilution were performed before and during TLV.

**Results:**

Left ventricular end diastolic pressure (LVEDP) (9.3 ± 2.1 vs. 9.2 ± 2.4mmHg, p = 0.89) and dimension (1.90 ± 0.09 vs. 1.86 ± 0.12cm, p = 0.72), negative dP/d*t* (-2589 ± 691 vs. -3115 ± 866mmHg/s, p = 0.50) and cardiac output (436 ± 28 vs. 481 ± 59ml/kg/min, p = 0.26) were not affected by TLV initiation. Left ventricular relaxation time constant (tau) slightly increased from 21.5 ± 3.3 to 24.9 ± 3.7ms (p = 0.03). Mean arterial systemic (48 ± 6 vs. 53 ± 7mmHg, p = 0.38) and pulmonary pressures (31.3 ± 2.5 vs. 30.4 ± 2.3mmHg, *p* = 0.61) were stable. As expected, the inspiratory phase of liquid cycling exhibited a small but significant effect on most variables (i.e. central venous pressure +2.6 ± 0.5mmHg, p = 0.001; LVEDP +1.18 ± 0.12mmHg, p<0.001).

**Conclusions:**

TLV was well tolerated in our neonatal lamb model of severe respiratory distress syndrome and had limited impact on left ventricle diastolic function when compared to conventional mechanical ventilation.

## Introduction

**Fig 1:**
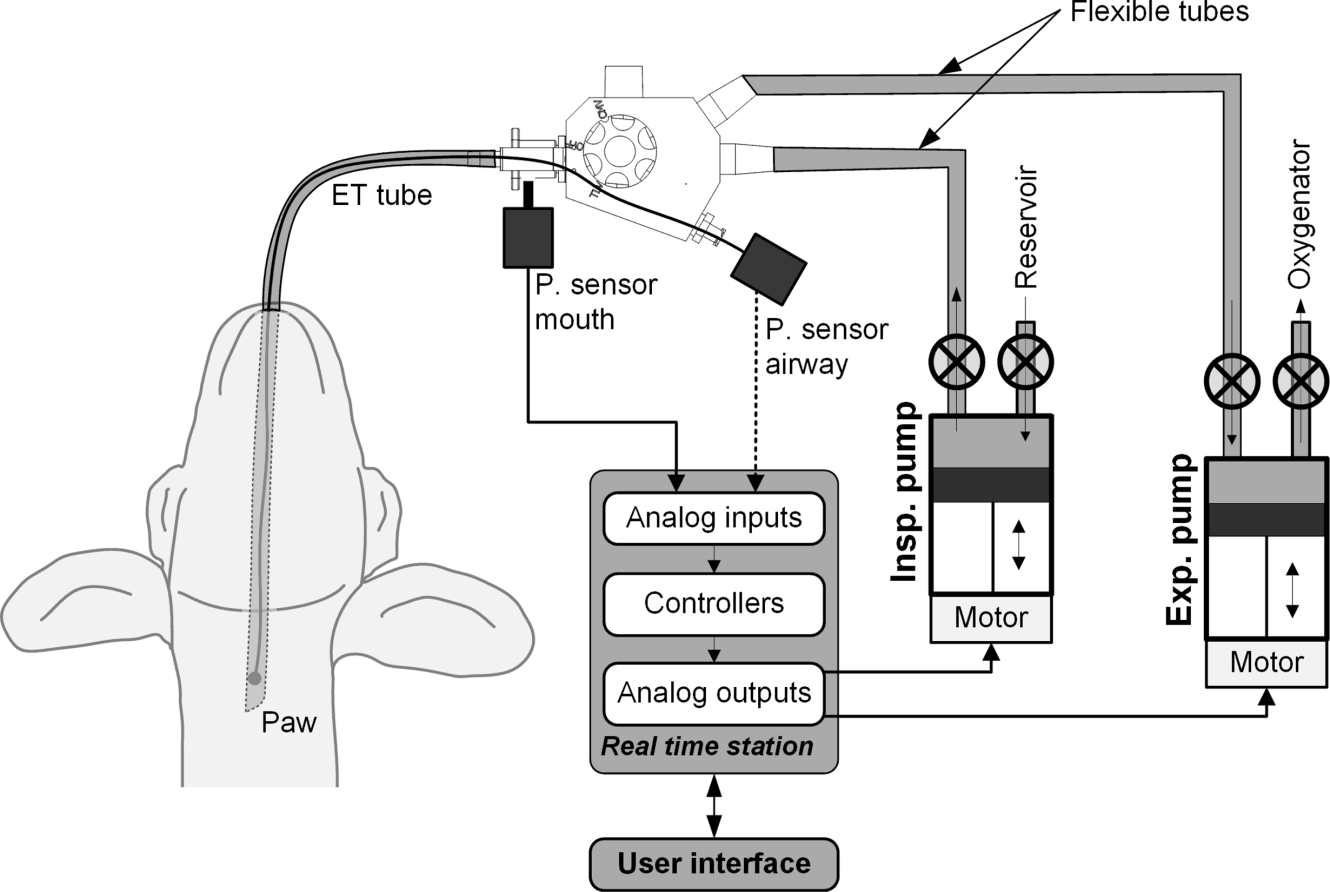
The INOLIVENT-6 liquid ventilator prototype. The inspiratory pump (Insp. Pump) delivers the tidal inspiratory volume of perflubron into the lungs. The expiratory pump (Exp. Pump) removes the tidal expiratory volume of perflubron from the lungs. The pressure sensor located at the mouth (P. sensor mouth) is used to limit the driving inspiratory and expiratory pressures and to monitor the pause pressure. P. sensor airway was previously used in the past to characterize the pressure drop although is no longer used.

Total liquid ventilation (TLV) uses liquid perfluorochemicals (PFC), known for their ability to dissolve high quantities of oxygen and carbon dioxide, in order to fill and ventilate the lungs using a dedicated liquid ventilator. This technique differs from partial liquid ventilation [1] since the lung is no longer subjected to conventional ventilation or to an air-liquid interface. Given that PFCs feature a very low surface tension and improve lung compliance [2, 3], TLV is appealing for the treatment of the premature infant suffering from respiratory distress syndrome due to lack of endogenous surfactant [4]. Unfortunately, the development of dedicated ventilators for TLV has been a challenge, and administration of poorly-controlled PFC volume has led to serious complications, such as lung overdistention and fluorothoraces [5]. Throughout the years, these ventilators have evolved from basic systems using gravity in order to fill and empty the lungs [6] to a dual-piston ventilator driven by complex ventilation algorithms [7–12]. The INOLIVENT prototype [8–12] (Fig 1) used for this study allows for the precise control over the volumes administered during TLV while continuously monitoring applied pressures.

As we progress from the proof of concept stage and envision human clinical trials, refinement of TLV ventilation techniques and safety assessment become increasingly important. Reports of the impact of TLV on hemodynamic stability have been inconsistent. Lowe et al. observed a 40% decrease in cardiac output (CO) during TLV [13]. The authors hypothesized that fluid-filled lungs by dense PFC (i.e. 1.93 g/ml for perflubron used in the present study) may compress the heart, great veins and aorta. Curtis et al., however, showed that increasing preload allowed the maintenance of CO for several hours during TLV [6]. We suspect that distinct TLV strategies, especially those related to the administered PFC volumes, differentially affect the various cardiovascular structures, just as for conventional ventilation approaches [14]. Indeed, the study by Lowe et al. featured higher liquid tidal volumes (22 ml/kg) than the volumes in the Curtis et al. study (approx. 16 ml/kg), with very high total PFC volume at end inspiration (75ml/kg in Lowes et al. while not reported in Curtis et al.), potentially explaining differences in hemodynamic responses. Moreover, high proximal inspiratory pressures must be generated by the ventilator in order to instill the viscous PFC into the lungs. Such pressure, although decreasing rapidly within the ventilator circuit and subsequently in the proximal airways, may contribute to the fluctuation documented in systemic arterial blood pressure [5, 7] and other hemodynamic variables. For example, using the INOLIVENT prototype, it was shown that for an inspiratory pressure of 90 cmH_2_O measured proximally to the endotracheal tube, the pressure at the distal end of the tube had already dropped to 30 cmH_2_O while ventilating with perflubron [15] (pressure measurement sites shown in Fig 1).

In light of the above, the objective of the present study was to assess the impacts of TLV on the cardiovascular system, in particular left ventricular (LV) diastolic function, in a neonatal lamb model on respiratory distress syndrome using the INOLIVENT prototype. We hypothesize that our current TLV approach, focusing on the use of the lowest possible volumes (i.e. tidal and total PFC volumes) will have very limited impact on hemodynamic stability and LV diastolic function.

## Material and methods

This experimental crossover study was conducted in eight term newborn male lambs 1 to 4 days of age. It is part of a larger project aimed at the assessment of TLV potential in treating respiratory distress syndrome of the newborn. This study was approved by the animal research ethics board of the Université de Sherbrooke (Protocol number: 401-16BR) and was designed in accordance with the Canadian Council on Animal Care guidelines.

### Anesthesia, instrumentation and induction of surfactant deficiency

Lambs were premedicated with ketamine (10 mg/kg, IM) prior to percutaneous left jugular vein cannulation. After intubation with a 4.5 mm cuffed endotracheal tube, lambs were placed in supine position and conventional mechanical ventilation was initiated (Servo-300; Siemens-Elema, Solna AB, Solna, Sweden) in a pressure regulated mode, with a positive end-expiratory pressure of 5 cmH_2_O and a peak inspiratory pressure adjusted to generate 7 ml/kg of tidal volume. Hemoglobin saturation was continuously recorded by pulse oximetry (Radical, Masimo, Irvine, CA). Lambs were maintained under general anesthesia during the entire experiment (propofol 120 mcg/kg/min, ketamine 1 mg/kg/h, along with a D10W at a rate of 6 ml/kg/h), and received a single dose of fentanyl 4 μg/kg before instrumentation. A 5 Fr Swan-Ganz catheter (132F5, Edwards Lifesciences, Irvine, CA) was inserted into the right external jugular vein through a cervical cutdown and advanced to the pulmonary artery for pressure measurements. A proximal port was used to monitor central venous pressure (CVP). A 3 Fr catheter (PV2013L07, PiCCO catheter; Pulsion Medical System, Munich, Germany) was inserted into the right femoral artery through a cutdown to allow for measurements of systemic arterial pressure and cardiac output using the thermodilution technique [16]. A high-fidelity 3.5 Fr microtip pressure catheter (SPR-524, Millar Instruments inc., Houston, TX) was introduced into the right carotid and advanced to the left ventricle (LV) for pressure measurements. Repeated lung lavages using 10 ml/kg of warmed saline were performed until PaO_2_ reached a value of < 100 torr under 100% FiO_2_ for at least 20 minutes. A decision was made prior to experimentations not to correct eventual acidosis in order to avoid masking progressive hemodynamic deterioration and poor perfusion. Following completion of the experiments, lambs were euthanized with an overdose of pentobarbital (100 mg/kg iv).

### TLV protocol

**Fig 2:**
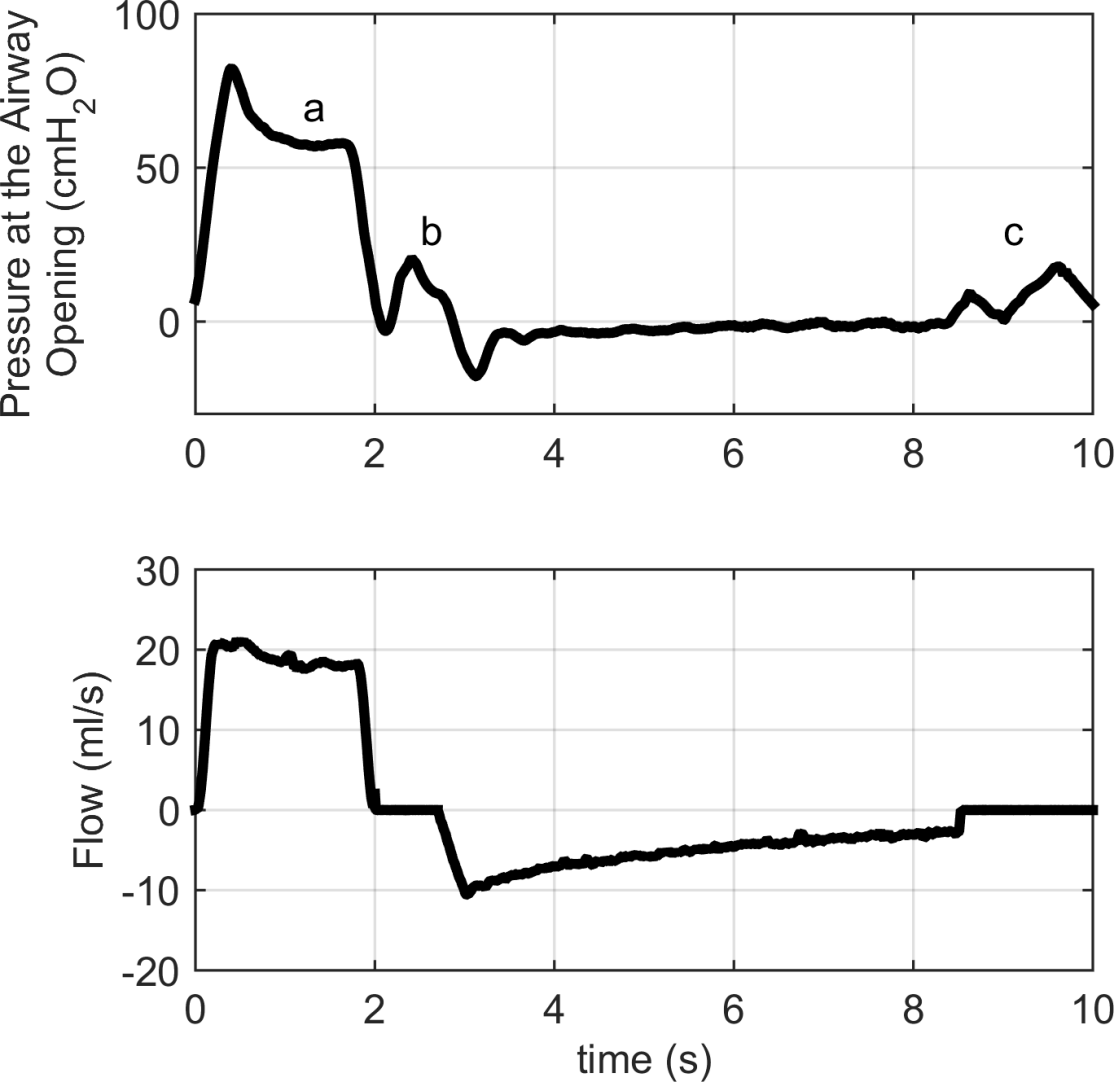
Pressure and flow during total liquid ventilation, measured upstream of the endotracheal tube. The pressure generated by the ventilator, while fluid is instilled into the lungs, is identified by the letter "a" on the upper panel. As the movement of PFC stops ("b" at end inspiration and "c" at end expiration), pressure oscillates around a value that can be extrapolated [18]. These values, called pause pressures, are the alveolar pressures at end inspiration and end expiration.

Lambs were disconnected from the gas ventilator for 5 seconds to allow for lung deflation (0 cmH_2_O positive end expiration pressure) prior to initiation of TLV. Lungs were then filled with 25 ml/kg of perflubron at 39°C (Exfluor, Round Rock, TX) over 18 sec, using the INOLIVENT-6 liquid ventilator prototype [9, 10]. End expiratory PFC volume was then increased progressively over the next 3–4 cycles to approximately 30 ml/kg in order to allow for air evacuation and limit total lung volume. Volume-controlled, pressure-limited and time-cycled TLV was then initiated aimed at the following parameters: tidal volume of 11–14 ml/kg, respiratory rate of 6–10 cycles/min and inspiratory/expiratory ratio of 1:2. End expiratory lung volume was carefully minimized to avoid expiratory tracheal collapses. Our ventilation approach has evolved over the years to aim for the lowest possible end expiratory lung volume as well as low tidal volume to limit volutrauma, a known contributor to ventilator-induced lung injury in the preterm infant. Consequently, ventilation rates used are at the higher end of what is usually reported for TLV in order to maintain sufficient minute ventilation. Ventilation parameters were set so as to maintain oxygen saturation levels above 90% and PaCO_2_ between 50–65 torr [17]. The INOLIVENT-6 prototype monitors both the inspiratory and expiratory pause pressures as well as the pressure during PFC movement in and out of the lungs (Fig 2). The pressure sensor is located immediately proximal to the junction between the ventilator and the endotracheal tube (Fig 1).

### Variables and statistical analysis

Central venous pressure (CVP), LV pressure, mean systemic arterial pressure (MSAP) and mean pulmonary arterial pressure (MPAP) were continuously recorded with AcqKnowledge (MP100, Biopac, Goleta, CA). Values were obtained just before disconnecting the subject from the conventional gas ventilator and 1 minute after initiation of TLV. LV end diastolic pressure (LVEDP, a diastolic function marker) was measured and both systolic (dP/d*t*) and diastolic (negative dP/dt, tau) function markers were generated by the software. Both positive and negative dP/d*t* are obtained from the derivative of the pressure curve. For tau calculation, the relaxation period is defined as the range of data between the time of minimum dP/dt in the cycle to the point where the LV pressure signal drops below the previous LVEDP level. Within this range, the following model is fitted to the data using the simplex search method:

$$P_{0}e^{t/\tau }$$

where *P*_0_ is the value of the left ventricular pressure signal at the time of dP/d*t* minimum and *t* is the time coordinate shifted such that *t* is 0 at the time of dP/d*t* minimum. The best fit value from this model is used as the value of the relaxation time constant. Variables derived from LV pressure recordings were averaged from 5 heart beats during the inspiratory as well as expiratory phases of TLV.

Shortening fraction and left ventricle end diastolic diameter were measured by echocardiography before and after 2 hours of TLV (M-Turbo Ultrasound System, Sonosite, Bothell, WA) using accepted guidelines [19]. M-mode was used on a parasternal short-axis view at the level of the tip of the mitral valve leaflets and averaged over 3 cardiac cycles. Due to the complexity of the protocol, echocardiographic measurements could not be performed immediately after the initiation of TLV but rather between 1 and 2 hours after filling. Cardiac output was measured before and after 2 hours of TLV using the transpulmonary thermodilution method (injection of 4 ml cold saline through the jugular vein and temperature assessment at the femoral arterial line, repeated 3 times for each timepoint) [16]. Arterial blood gases were measured at each phase of the experiment (Rapidlab 348, Siemens, AB, Solna, Sweden). Data are presented as mean ± SEM. Paired t-test was used for comparisons. All statistical analyses were performed using SPSS 19 software (IBM, North Castle, NY). A value of < 0.05 was deemed to be statistically significant.

## Results

Eight lambs aged 3.0 ± 0.4 days of life and weighing 3.2 ± 0.3 kg were used. Lambs reached entry criteria after 9 ± 3 lung lavages. The animals presented a severe respiratory distress after the insult and the peak inspiratory pressure had to be increased to maintain tidal volume (15.0 ± 0.6 vs. 20.9 ± 1.3 cmH_2_O, p < 0.001). pH decreased from 7.31 ± 0.04 to 7.12 ± 0.03 (*p* < 0.001), PCO_2_ increased from 42 ± 2 to 61 ± 2 torr (*p* = 0.001), PaO_2_/FiO_2_ decreased from 297 ± 37 to 70 ± 7 (p < 0.001) and HCO3 decreased from 20 ± 2 to 16 ± 1 mmol/l (*p* = 0.02). Blood pressure also decreased significantly from 68 ± 8 to 48 ± 6 mmHg (p = 0.03). The lungs were thereafter filled with PFC followed by initiation of ventilation using tidal volumes of 12.6 ± 0.4 ml/kg at a rate of 6.5 ± 0.2 cycles/min. After 1 minute of TLV, PFC end expiratory volume was stabilized at 29.1 ± 0.5 ml/kg.

### Effect of TLV initiation on LV diastolic function and hemodynamic stability

**Fig 3:**
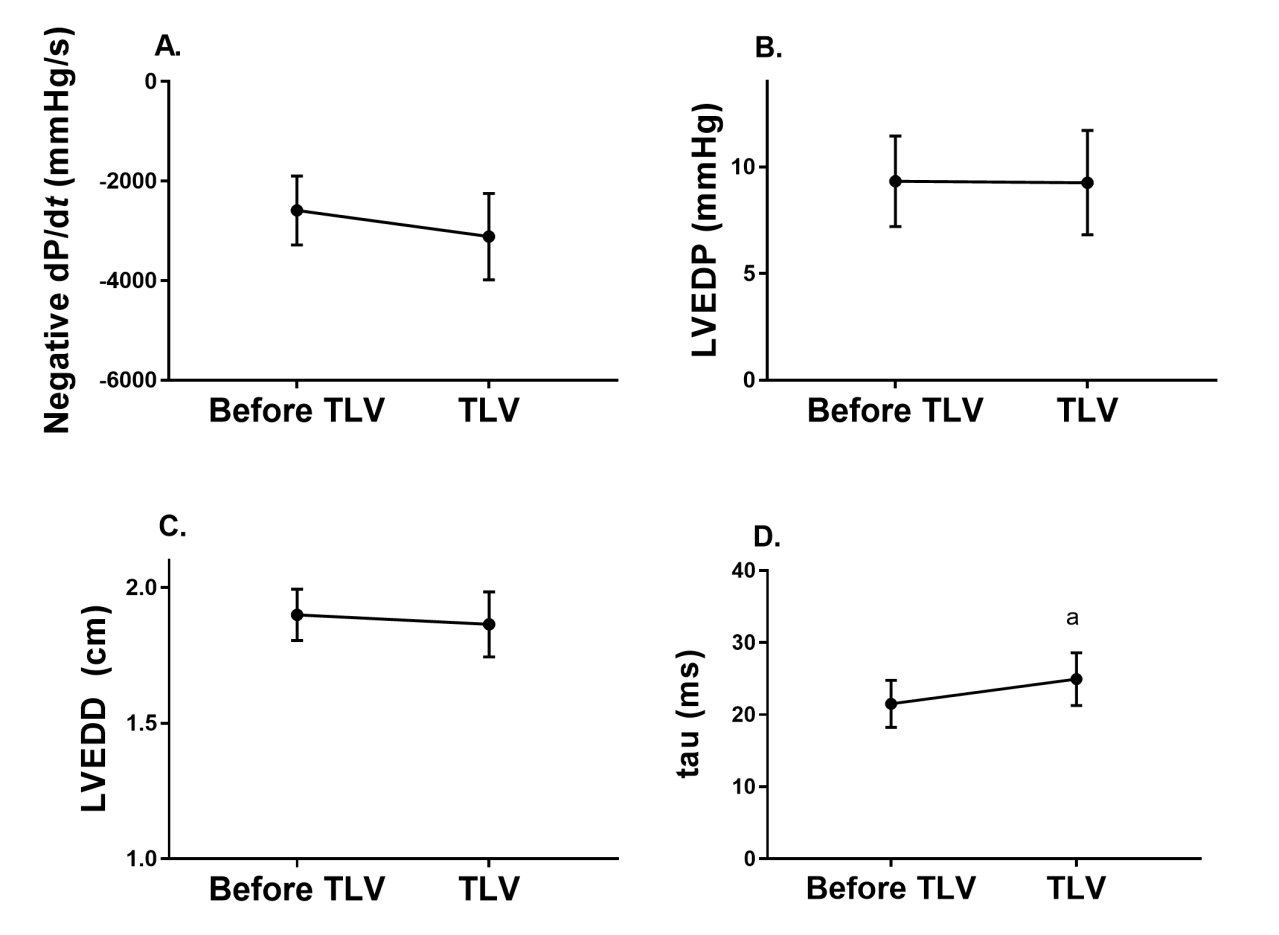
Impact of total liquid ventilation on left ventricle diastolic function. A. Negative dP/d*t*; B. Left ventricular end diastolic pressure; C. Left ventricular end diastolic dimension; D. Left ventricular relaxation time constant (tau). ^a^p < 0.05.

TLV did not affect LV end diastolic pressure or negative dP/d*t* (Fig 3). However, there was a small effect on LV relaxation time constant (tau), from 21.5 ± 3.3 to 24.9 ± 3.7 ms (*p* = 0.025). Assessment of LV end diastolic dimension (LVEDD) and cardiac output performed between 1 and 2 hours of TLV revealed that LVEDD was preserved (Fig 3), as was cardiac output (436 ± 28 vs. 481 ± 59 ml/kg/min, *p* = 0.26) when compared to post-insult values (i.e. immediately prior to TLV).

TLV was well tolerated. There was a small increase in CVP which did not reach statistical significance (8.5 ± 2.7 vs. 10.0 ± 2.8 mmHg after filling, *p* = 0.06) whereas shortening fraction (28.5 ± 2.3 vs. 30.4 ± 1.3%, *p* = 0.36), positive dP/dt (2486 ± 691 vs. 2181 ± 467 mmHg/s, p = 0.65), MPAP (31.3 ± 2.5 vs. 30.4 ± 2.3 mmHg, *p* = 0.61) and MSAP (48 ± 6 vs. 53 ± 7 mmHg, *p* = 0.38) were all preserved.

### Effect of perflubron cycling

**Fig 4:**
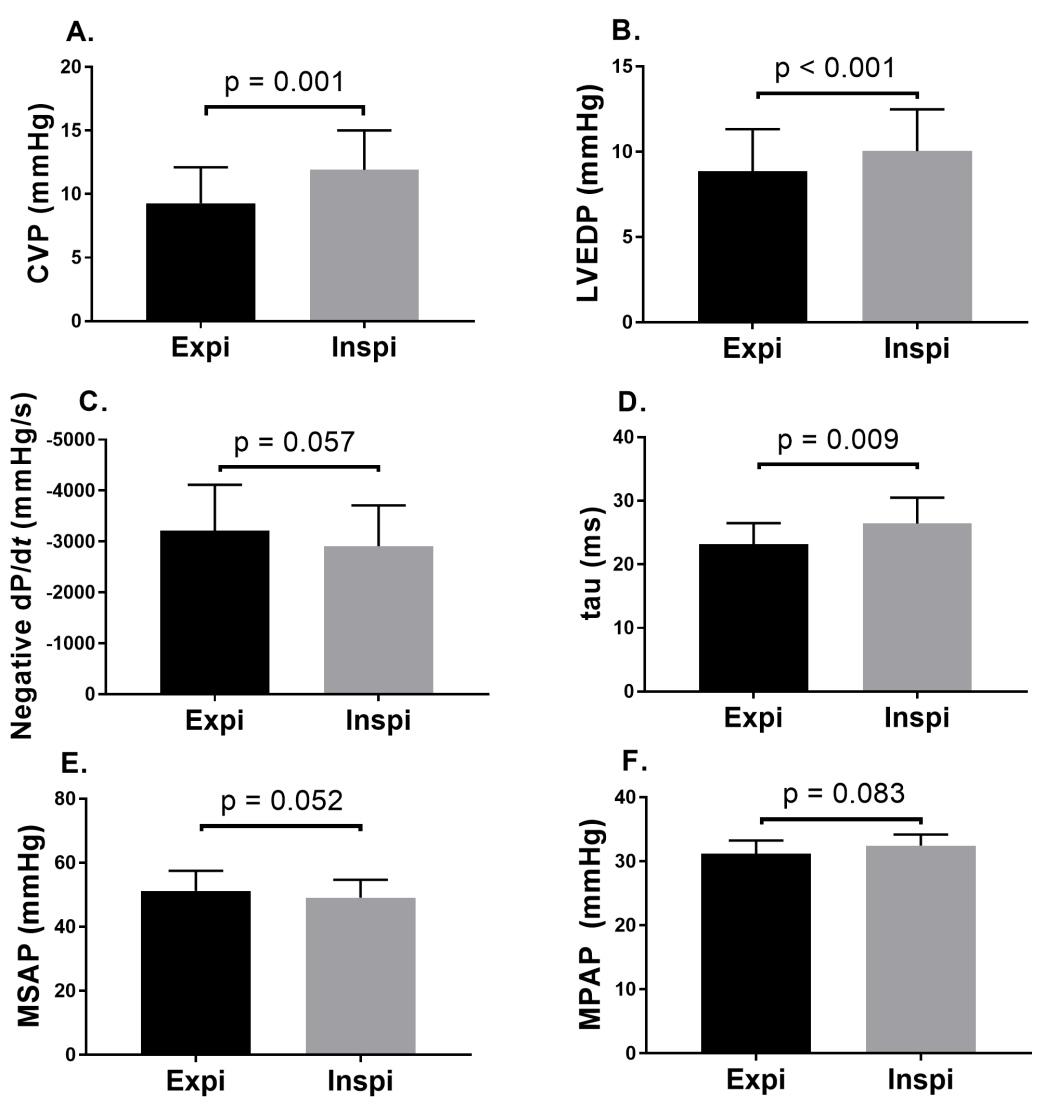
Hemodynamic impact of perflubron cycling. A. central venous pressure; B. Left ventricular end diastolic pressure; C. Negative dP/d*t*; D. Left ventricular relaxation time constant (tau); E. Mean systemic arterial pressure; F. Mean pulmonary arterial pressure.

Initial inspiratory pause pressure was measured at 18 ± 1 cmH_2_O and end expiratory pause pressure at 12 ± 1 cmH_2_O (Fig 2). Both these pressures decreased within the first minutes of TLV (15 ± 1 and 4 ± 1, *p* = 0.002 and *p* < 0.001, respectively, after 30 minutes of TLV). The inspiratory pressure (i.e. during fluid movement) measured proximal to the endotracheal tube was 54 ± 5 cmH_2_O at the time of the recordings. Fig 4 shows the effects of TLV cycling (inspiration and expiration phases) on CVP, LEVDP, negative dP/d*t*, tau, MSAP and MPAP. The inspiration phase was associated with an increase in CVP (*p* = 0.001), LVEDP (*p* < 0.001) and tau (*p* = 0.009). However, MSAP and MPAP were not affected.

### Tolerance to the protocol

Lambs received 3.0 ± 1.1 bolus of 10 ml/kg normal saline during anesthesia (415 ± 25 min). MSAP remained in acceptable range during the TLV (53 ± 7mmHg after initiation of TLV vs. 51 ± 6 at 2 hours, *p* = 0.71) while blood gases gradually stabilized between the insult and 2 hours into the TLV protocol (pH 7.12 ± 3 vs. 7.15 ± 3, *p* = 0.06; PaO_2_ 70 ± 7 vs. 131 ± 13 torr, *p* = 0.003; PCO_2_ 61 ± 2 vs. 57 ± 1 torr, *p* = 0.18; HCO_3_ 16.2 ± 0.8 vs. 16.9 ± 0.9 mmol/l *p* = 0.06).

## Discussion

Reported hemodynamic impacts of TLV have been inconsistent [5–7, 20]. Using the INOLIVENT prototype with a well-defined ventilation strategy, TLV was found to be well tolerated overall in our neonatal lamb model of severe respiratory distress syndrome. Results demonstrated only a subtle effect on LV diastolic function without significant effect on hemodynamic stability. These effects were mostly observed during the inspiratory phase, albeit of limited amplitude. Although work remains to be done, we believe TLV could be used safely to treat respiratory distress syndrome in the most extreme premature neonates (i.e.22-24 weeks gestation) in order to prevent ventilator-induced lung damage and subsequent bronchopulmonary dysplasia.

In 1979, Lowe et al. had hypothesized that the hemodynamic instability documented with TLV could be secondary to direct compression of the myocardium by the liquid-filled lung [13], impeding LV diastolic filling. In the present study, initiation of TLV had no impact on negative dP/d*t* while tau only increased very mildly. Of further importance, LVEDP did not increase with TLV and there was no immediate effect on blood pressure, thus suggesting preserved LV filling and cardiac output. Subsequent echocardiographic assessment demonstrated good ventricular filling and thermodilution confirmed preserved cardiac output. At most, only subtle changes during the inspiration phase of TLV could be documented.

Fluctuations in cardiac output as well as arterial blood pressure with respiratory cycle during mechanical ventilation have been reported in both gaseous ventilation [21] and TLV [5, 7]. In the present study, the inspiration phase had an impact on most monitored variables, although the differences were of limited amplitude. This latter finding strengthens a long-held belief within our team that total volume of PFC within the lungs must be tightly regulated. A recent study also suggested the use of lower volumes to prevent lung inflammation during TLV [22], in keeping with the overwhelming evidence that volutrauma is a significant contributor to ventilator-induced lung injury during conventional mechanical ventilation [23]. Thus, we hypothesize that uncontrolled volumes of PFC within the lung and/or high pause inspiratory and expiratory pressures may affect cardiac function, which may explain earlier findings of instability with less advanced TLV devices. Our results are in agreement with Tsagogiorgas et al. showing no significant effect of TLV on cardiac output, pulmonary vascular resistance and left atrial pressure in a healthy rabbit model [7] using a dedicated ventilator. However, direct measurements of LV diastolic function (tau, negative dP/dt and LVEDD) had not been assessed in this latter study which was performed in healthy mature subjects. A study by Degraeuwe [5], also using a neonatal model of surfactant deficiency, had demonstrated significant variation in hemodynamic parameters with PFC cycling. However, the ventilator used was less sophisticated and end expiratory lung volumes could not be controlled, leading to 3 fluorothoraces and subsequent death of the animals. By tightly matching expiratory to inspiratory PFC volume during cycling, through avoidance of tracheal collapse during expiration [11, 24], end expiratory lung volume can be maintained to a minimal level and thus prevent the development of such complications. Although there is no consensus on specific parameters to be used during TLV, we believe limiting lung volume is the safest way forward and should be carefully considered when this technology will be used in a clinical trial.

### Limitations

Our model had significant multifactorial mixed acidosis that could have affected hemodynamic stability. This acidosis was present prior to PFC filling and improved during TLV, thus we are confident there is no causal relationship between TLV and acidosis. The severity of the hypoxic insult, the use of propofol and restriction of blood flow to the leg secondary to the femoral catheter were likely contributors. Acidosis could have rendered the animals more fragile during TLV. However, given the absence of hemodynamic instability (i.e. the animals maintained cardiac output, blood pressure and diuresis), we are confident in our conclusions. Continuous recording from invasive devices allowed conducting measurements both immediately before and after initiation of TLV, isolating the latter from confounding factors such as prolonged anesthesia, intravenous infusion of normal saline and pH variation. Unfortunately, cardiac output measurements by thermodilution and echocardiographic studies could not be conducted immediately after filling due to technical reasons. One could assume that these two measurements may have potentially been affected. However, since TLV had very limited immediate effect on both blood pressure and invasive hemodynamic variables, we are once again confident in our general conclusion of good hemodynamic tolerance of TLV.

## Conclusion

This study demonstrates that TLV does not significantly impede LV filling in a model of neonatal surfactant deficiency using our dedicated ventilator with proper ventilation algorithms and a strategy based on controlling lung PFC volume. The lack of standards in liquid ventilation technologies may explain the inconsistency in previously reported data.
